# BST2 Suppresses LINE-1 Retrotransposition by Reducing the Promoter Activity of LINE-1 5′ UTR

**DOI:** 10.1128/JVI.01610-21

**Published:** 2022-01-26

**Authors:** Yifei Zhao, Juan Du, Yu Wang, Qing Wang, Shaohua Wang, Ke Zhao

**Affiliations:** a Institute of Virology and AIDS Research, First Hospital of Jilin University, Changchun, China; b Center for Pathogen Biology and Infectious Diseases, First Hospital of Jilin University, Changchun, China; c Key Laboratory of Organ Regeneration & Transplantation of the Ministry of Education, First Hospital of Jilin University, Changchun, China; d Department of Respiratory Medicine, First Hospital of Jilin University, Changchun, China; Icahn School of Medicine at Mount Sinai

**Keywords:** BST2, LINE-1, genome stabilization, innate immune regulation, promoter regulation, retrotransposon

## Abstract

Endogenous retrotransposons are considered the “molecular fossils” of ancient retroviral insertions. Several studies have indicated that host factors restrict both retroviruses and retrotransposons through different mechanisms. Type 1 long interspersed elements (LINE-1 or L1) are the only active retroelements that can replicate autonomously in the human genome. A recent study reported that LINE-1 retrotransposition is potently suppressed by BST2, a host restriction factor that prevents viral release mainly by physically tethering enveloped virions (such as HIV) to the surface of producer cells. However, no endoplasmic membrane structure has been associated with LINE-1 replication, suggesting that BST2 may utilize a distinct mechanism to suppress LINE-1. In this study, we showed that BST2 is a potent LINE-1 suppressor. Further investigations suggested that BST2 reduces the promoter activity of LINE-1 5′ untranslated region (UTR) and lowers the levels of LINE-1 RNA, proteins, and events during LINE-1 retrotransposition. Surprisingly, although BST2 apparently uses different mechanisms against HIV and LINE-1, two membrane-associated domains that are essential for BST2-mediated HIV tethering also proved important for BST2-induced inhibition of LINE-1 5′ UTR. Additionally, by suppressing LINE-1, BST2 prevented LINE-1-induced genomic DNA damage and innate immune activation. Taken together, our data uncovered the mechanism of BST2-mediated LINE-1 suppression and revealed new roles of BST2 as a promoter regulator, genome stabilizer, and innate immune suppressor.

**IMPORTANCE** BST2 is a potent antiviral protein that suppresses the release of several enveloped viruses, mainly by tethering the envelope of newly synthesized virions and restraining them on the surface of producer cells. In mammalian cells, there are numerous DNA elements replicating through reverse transcription, among which LINE-1 is the only retroelement that can replicate autonomously. Although LINE-1 retrotransposition does not involve the participation of a membrane structure, BST2 has been reported as an efficient LINE-1 suppressor, suggesting a different mechanism for BST2-mediated LINE-1 inhibition and a new function for BST2 itself. We found that BST2 specifically represses the promoter activity of LINE-1 5′ UTR, resulting in decreased levels of LINE-1 transcription, translation, and subsequent retrotransposition. Additionally, by suppressing LINE-1 activity, BST2 maintains genome stability and regulates innate immune activation. These findings expand our understanding of BST2 and its biological significance.

## INTRODUCTION

Among transposable elements that constitute over 40% of the human genome, only type 1 long interspersed elements (LINE-1 or L1) replicate autonomously (1). A typical LINE-1 DNA is approximately 6-kb long and contains two open reading frames (ORFs), *orf1* and *orf2*, which are flanked by 5′- and 3′-untranslated regions (UTRs). The 5′ UTR functions as a promoter in its DNA form and provides a ribosome binding site similar to that of its RNA (2), whereas the 3′ UTR stimulates LINE-1 retrotransposition (3). The proteins expressed from these ORFs, namely, ORF1p and ORF2p, are both RNA-binding proteins that preferentially interact with the LINE-1 RNA from which they are expressed (4). In addition, ORF2p performs endonuclease and reverse transcriptase activities that are essential not only for LINE-1 retrotransposition (5, 6) but also for the replication of other active yet nonautonomous retroelements, such as Alu and SVA (7, 8). The binding between ORF1p, ORF2p, and LINE-1 RNA triggers the assembly of additional cellular factors to form LINE-1 ribonucleoprotein particles (RNPs), which are the fundamental units of LINE-1 retrotransposition (9, 10).

Previous studies have revealed the long-term effects of LINE-1 on genome evolution, such as epigenetic regulation, 5′ and 3′ transduction, exon skipping, transcription termination, and gene breaking (11, 12). However, recent efforts have focused on its short-term effects. For instance, it has been determined that if out of control, LINE-1 induces DNA damage in the host genome, resulting in the disruption of cell cycle (13, 14). Further studies have suggested and/or demonstrated that LINE-1 (and its replication) is an endogenous trigger of the innate immune system (15, 16). However, it is noteworthy that LINE-1-mediated innate immune activation is delicate because it renders the host vulnerable to exogenous pathogens or development of autoimmune diseases, depending on the level of interferons (IFNs) produced based on the activation of the innate immune system (16). Accordingly, despite the fact that only 80–120 copies out of ∼500,000 copies of LINE-1 remain retrotransposition competent per single human cell, LINE-1 retrotransposition must be carefully regulated.

Several host factors in human cells have been confirmed to possess anti-LINE-1 activity. Interestingly, many of these factors also target retroviruses, such as HIV-1. However, this is not because of similarities between retroelements and retroviruses in their DNA, RNA, proteins, or replication processes. In fact, two or more different mechanisms are usually used by a single host factor to suppress LINE-1 and HIV-1. The most studied examples of such factors include members of the APOBEC3 family. These proteins have a deaminase function that introduces a G-to-A mutation in a newly synthesized HIV cDNA (17, 18). However, most APOBEC3 proteins suppress LINE-1 through deaminase-independent mechanisms (19). In addition, SAMHD1 suppresses HIV-1 in nondividing cells by reducing cellular dNTP levels (20, 21), whereas it inhibits LINE-1 replication in dividing cells by reducing the level of ORF2p and restricting the subcellular distribution of ORF1p (22, 23). Further, TREX1 prevents HIV integration by digesting reverse-transcribed viral cDNA (24); however, it protects the host genome from LINE-1 by inducing proteasomal degradation of ORF1p (25). Thus, these phenomena indicate that, although similar in many aspects, retroviruses and retroelements may impose different selection pressures on host factors.

Interestingly, a host restriction factor that has been confirmed to suppress LINE-1 replication is the bone marrow stromal cell antigen 2 (BST2) (26). BST2 is a type-II transmembrane glycoprotein capable of tethering enveloped virions, including HIV-1 and hepatitis B virus (HBV), at the cell surface (hence the protein is also known as tetherin) (27–29). BST2 contains several domains, including a cytoplasmic tail (CT), a transmembrane region (TM), an extracellular coiled-coil domain (CC), and a glycosyl-phosphatidylinositol (GPI) anchor covalently linked to the membrane (30). It is well accepted that, during viral release, one end of BST2 (i.e., TM or GPI) is attached to the viral membrane, while the other end (i.e., GPI or TM) remains associated with the cytoplasmic membrane, thus trapping the virions outside the host cell (27, 31). CC is also critical for the antiviral activity of BST2, as it induces the formation of a functional BST2 dimer (32). CT activates the NF-κB-dependent proinflammatory response, which also contributes to HIV suppression (33, 34). Otherwise, CT is important for the BST2-MAVS interaction, which promotes the autophagic degradation of MAVS and negatively regulates the type 1 IFN signaling pathway by recruiting E3 ubiquitin ligase MARCH8 (35).

However, to our knowledge, the replication of LINE-1 does not involve any activity on either the cytoplasmic or endoplasmic membrane. It is, therefore, highly possible that BST2 inhibits LINE-1 retrotransposition through a different mechanism. In this study, we confirmed that BST2 is a potent LINE-1 suppressor. Surprisingly, neither CT nor the CC domain was essential for BST2-mediated LINE-1 suppression; instead, this BST2 function involved both membrane-associated domains (TM and GPI). Further investigations indicated that BST2 potently reduced the promoter activity of LINE-1 5′ UTR, resulting in decreased levels of LINE-1 RNA, proteins, and events during LINE-1 retrotransposition. Through LINE-1 inhibition, BST2 also suppressed LINE-1-induced genomic DNA damage and innate immune activation. Therefore, by investigating the mechanism of BST2-mediated LINE-1 suppression, our data revealed several new functions of BST2, including promoter regulation, genome stabilization, and innate immune suppression.

## RESULTS

### BST2 functions as a potent LINE-1 suppressor.

**FIG 1:**
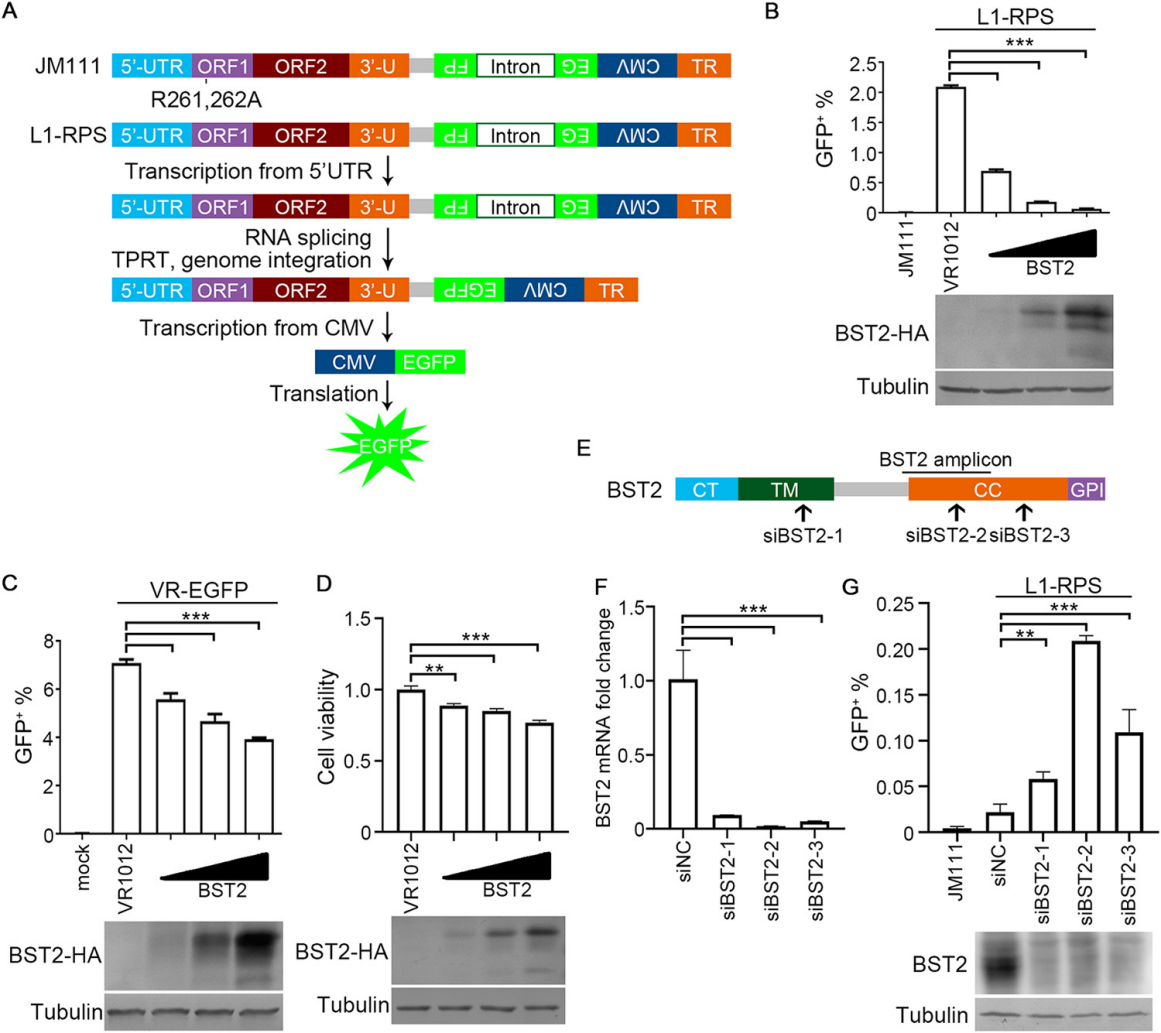
BST2 potently reduces LINE-1 retrotransposition activity. (A) Schematic of retrotransposition-competent LINE-1 plasmid 99 PUR RPS EGFP (L1-RPS) and its negative control JM111 EGFP (JM111), and the pattern of L1-RPS retrotransposition as described previously (25). The antisense *EGFP* gene could not be translated directly due to an interruption by a sense intron. EGFP can only be expressed when L1-RPS is transcribed, spliced (to remove the intron), reverse transcribed, and integrated into the host genome. The R261A/R262A mutation in ORF1p abolishes the retrotransposition potency of JM111, which was used as a negative control. (B) LINE-1 assay results indicating that BST2 suppresses LINE-1 activity in a dosage-dependent manner in HEK293T cells. (C) BST2 is much less effective in suppressing CMV promoted-EGFP reporter expression in HEK293T cells. (D) BST2 is much less effective in suppressing cell viability. (E) Diagram showing the target sites of *BST2*-specific siRNA and the region of qRT-PCR amplicon based on *BST2*-specific primers. CT, cytoplasmic tail; TM, transmembrane region; CC, extracellular coiled-coil domain; GPI, glycosyl-phosphatidlyinositol anchor. (F) Endogenous mRNA level of BST2 tested using qRT-PCR in HeLa cells transfected with siRNA. (G) LINE-1 activity was potently increased upon siRNA treatment in HeLa cells. **, *p* < 0.01; ***, *p* < 0.001.

To confirm the potency of BST2 against LINE-1 activity, we used the widely accepted EGFP-based LINE-1 retrotransposition assay, which includes the use of retrotransposition-competent 99 PUR RPS EGFP (L1-RPS) and its negative control, 99 PUR JM111 EGFP (JM111; see Materials and Methods for more details) (36) (Fig. 1A). Exogenous expression of BST2 in HEK293T cells potently reduced the EGFP signal, an active marker of a post-retrotransposition event generated by L1-RPS (Fig. 1B). Further investigations indicated that BST2 mildly suppressed EGFP expression driven by a CMV promoter (Fig. 1C); however, this could not explain the above reduction of EGFP (whose expression is also driven by a CMV promoter) in the LINE-1 retrotransposition assay. Additionally, exogenous BST2 reduced cell proliferation to a mild degree (Fig. 1D), suggesting that BST2-mediated LINE-1 suppression was not due to the cytotoxicity induced by the protein. All these data were consistent with those of a previous study supporting the function of BST2 as a potent LINE-1 suppressor (26).

Further, to determine whether endogenous BST2 inhibits LINE-1 replication, we repeated the LINE-1 retrotransposition assay in HeLa cells, which, unlike HEK293T cells, express endogenous BST2 (29). BST2-specific siRNAs (siBST2) were designed, synthesized, and tested in the assay (Fig. 1E). All three siBST2 effectively reduced the endogenous expression of BST2 (Fig. 1F and G), while the activity of L1-RPS was elevated in siBST2-transfected HeLa cells (Fig. 1G). Thus, we confirmed that BST2 potently suppressed LINE-1 replication.

### BST2-mediates HIV and LINE-1 suppression through distinct mechanisms.

BST2 suppresses HIV (and some other enveloped viruses) mostly by tethering the progeny virions to the cytoplasmic membrane, thus preventing them from being released (27, 32). To our knowledge, replication of LINE-1 does not involve either cytoplasmic or endoplasmic membranes. Therefore, it is reasonable to speculate that BST2 utilizes a different mechanism to suppress LINE-1 expression.

**FIG 2:**
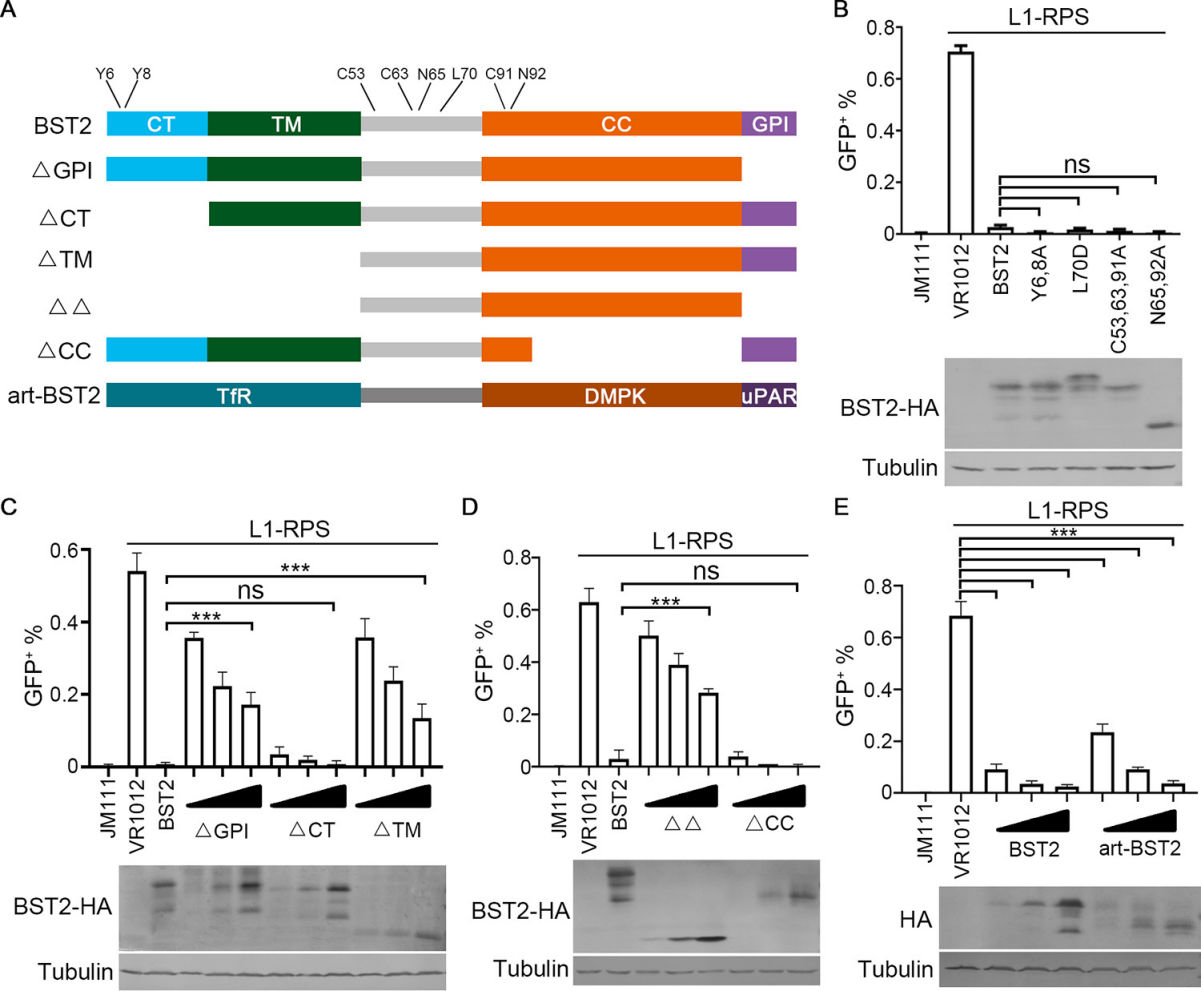
GPI and TM domains are important for BST2-mediated LINE-1 suppression. (A) Schematic of BST2 mutation, truncation constructs, and art-BST2 used in this study. The reconstruction of art-BST2 vector was based on a previous report, and the expressed protein contains N terminus from the transferrin receptor (TfR), coiled coil from dystrophia myotonica protein kinase (DMPK), and C terminus from urokinase plasminogen activator receptor (uPAR). (B) Points mutations reported to compromise the antiviral ability of BST2 do not affect BST2-mediated LINE-1 suppression in HEK293T cells. (C and D) Both GPI and TM domains are important for BST2-mediated LINE-1 suppression in HEK293T cells. (E) Art-BST2 suppresses LINE-1 activity in a dosage-dependent manner in HEK293T cells. ns, *p* > 0.05; ***p < 0.001.

To test this hypothesis, we first introduced several point mutations in BST2 that have been confirmed to compromise, if not abolish, the efficacy of BST2 against HIV (Fig. 2A). The BST2 residues C53, C63, and C91 form disulfide bonds that are important for BST2 dimerization (31); N65 and N92 are critical sites for BST2 glycosylation (31); L70 triggers the formation of BST2 homotetramers (24); and Y6 and Y8 are essential for BST2-mediated NF-κB activation (33). All these processes and functions contribute to the suppression of HIV to various degrees. Interestingly, none of the mutations at any of these positions affected the potency of BST2 in suppressing LINE-1 expression (Fig. 2B). Thus, the mechanism through which BST2 suppresses HIV does not play a role in BST2-mediated LINE-1 suppression.

In addition, BST2 contains several domains, most of which are essential for HIV suppression (27, 31, 37). To identify the domain(s) responsible for LINE-1 inhibition, we introduced deletions and truncations in BST2 (Fig. 2A). Removing CT did not affect the potency of BST2 against LINE-1 (Fig. 2C), which was surprising because it is the only cytoplasmic domain of BST2. Combined with previous results using the Y6, 8A mutant, we also confirmed that BST2-induced NF-κB activation is not involved in BST2-mediated LINE-1 suppression. Interestingly, the deletion of either TM or GPI compromised BST2-mediated LINE-1 regulation (Fig. 2C), and removing the membrane-associated regions (ΔΔ) almost completely abolished the efficiency of BST2 against LINE-1 retrotransposition (Fig. 2D). On the other hand, internal truncation of CC showed no effect on BST2-mediated LINE-1 suppression (Fig. 2D), confirming that the dimerization of BST2 is not required for LINE-1 inhibition, unlike in BST2-mediated trapping of HIV. Therefore, although different mechanisms are involved in BST2-mediated HIV and LINE-1 suppression, the membrane-associated regions of BST2 are required for LINE-1 regulation.

The above observations were surprising because, despite being essential in BST2-mediated viral suppression, neither TM nor GPI demonstrated additional biological functions other than maintaining BST2 on the membrane. Thus, we wondered whether suppressing LINE-1 requires specific amino acids on both domains or could be achieved with other domains with similar structures but different origins. It is noteworthy that an artificial BST2 designed by combining similar regions from different proteins showed potency in HIV inhibition (31). In this study, we reconstructed an artificial BST2 (art-BST2) expressing vector based on strategies previously employed and tested it in the LINE-1 retrotransposition assay (Fig. 2A). Interestingly, although distinct in protein sequence, art-BST2 showed similar efficiency in LINE-1 regulation compared with that of wild type BST2 (Fig. 2E). Thus, it appears that BST2 suppresses LINE-1 retrotransposition through a structure-dependent mechanism.

### BST2 reduces the levels of LINE-1 proteins (ORF1p and ORF2p).

**FIG 3:**
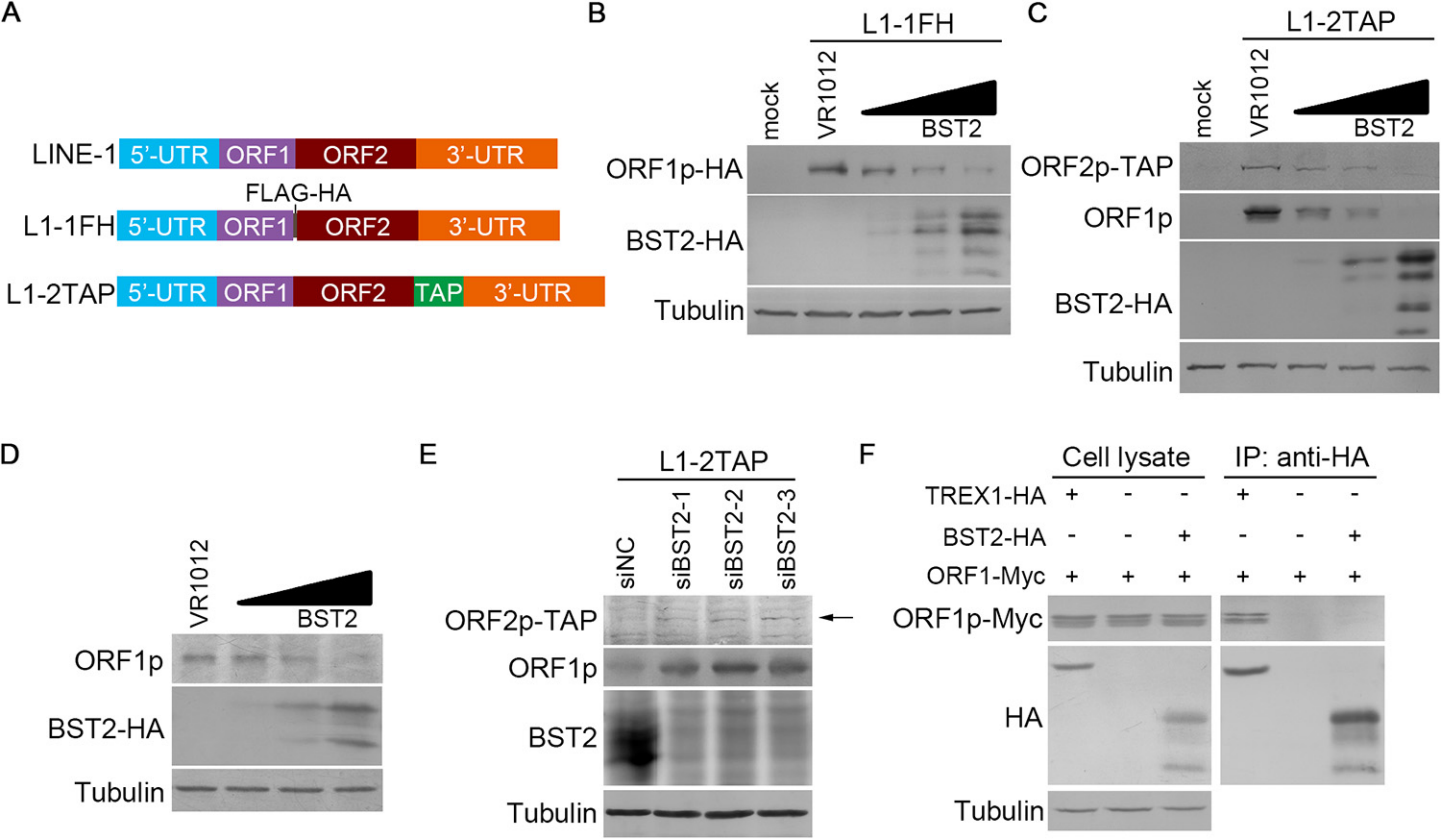
BST2 decreases the levels of LINE-1 proteins. (A) Schematic of LINE-1 expression vectors pc-L1-1FH (L1-1FH) and pc-L1-2TAP (L1-2TAP). ORF1p was tagged with both FLAG and HA tags in L1-1FH, while ORF2p was tagged with a tandem affinity purification (TAP) tag in L1-2TAP. (B) BST2 reduces ORF1p levels expressed from L1-1FH in HEK293T cells. (C) BST2 reduces both ORF1p and ORF2p levels expressed from L1-2TAP in HEK293T cells. (D) BST2 reduces endogenous ORF1p level in HEK293T cells. (E) Reduction of endogenous BST2 expression increases the levels of LINE-1 proteins in HeLa cells. (F) BST2 does not interact with ORF1p. TREX1 was used as positive control for ORF1p interaction (25).

Our data suggest that the two membrane-associated regions (i.e., TM and GPI) of BST2 are involved in BST2-mediated suppression of LINE-1 retrotransposition. First, we checked whether BST2 affects the expression and/or stability of LINE-1 proteins using L1-1FH, a LINE-1 construct that expresses ORF1p with a FLAG and an HA tag fused to its C-terminus (Fig. 3A). Co-transfection of L1-1FH and BST2 expression vectors into HEK293T cells revealed that BST2 potently reduces the protein levels of ORF1p (Fig. 3B). Afterward, we used the L1-2TAP construct that expresses ORF2p with a tandem affinity purification (TAP) tag at its C-terminus (Fig. 3A). Exogenous BST2 expression in HEK293T cells resulted in the reduction of ORF2p level (Fig. 3C). ORF1p properties may be altered by the presence of a fused tag (38). Notably, the level of untagged ORF1p expressed from L1-2TAP (Fig. 3C) as well as that of endogenous ORF1p in parallel experiments (Fig. 3D) was reduced by BST2, thereby excluding the possibility that the ORF1p reduction observed in Fig. 3B was due to the fused FLAG-HA tag. In contrast, reducing the endogenous expression of BST2 in HeLa cells promoted the expression of both ORF1p and ORF2p from L1-2TAP (Fig. 3E), confirming that BST2 potently affects the protein levels of ORF1p and ORF2p.

Subsequently, we sought to determine the mechanism by which BST2 decreases the levels of LINE-1 proteins, focusing on BST2-mediated ORF1p reduction. Protein–protein interaction is a common mechanism involved in the degradation of a protein by another, as previously observed in TREX1-mediated ORF1p depletion (25). Therefore, co-immunoprecipitation (co-IP) assays were performed to determine whether BST2 interacts with ORF1p, using TREX1 as a positive control. Results revealed a readily detectable interaction between TREX1 and ORF1p; however, BST2–ORF1p interaction was not detected (Fig. 3F), suggesting that BST2 inhibits the expression of ORF1p without making a direct contact and is thus unlikely through a post-translational mechanism.

### BST2 decreases LINE-1 RNA levels by suppressing the promoter activity of LINE-1 5′ UTR.

**FIG 4:**
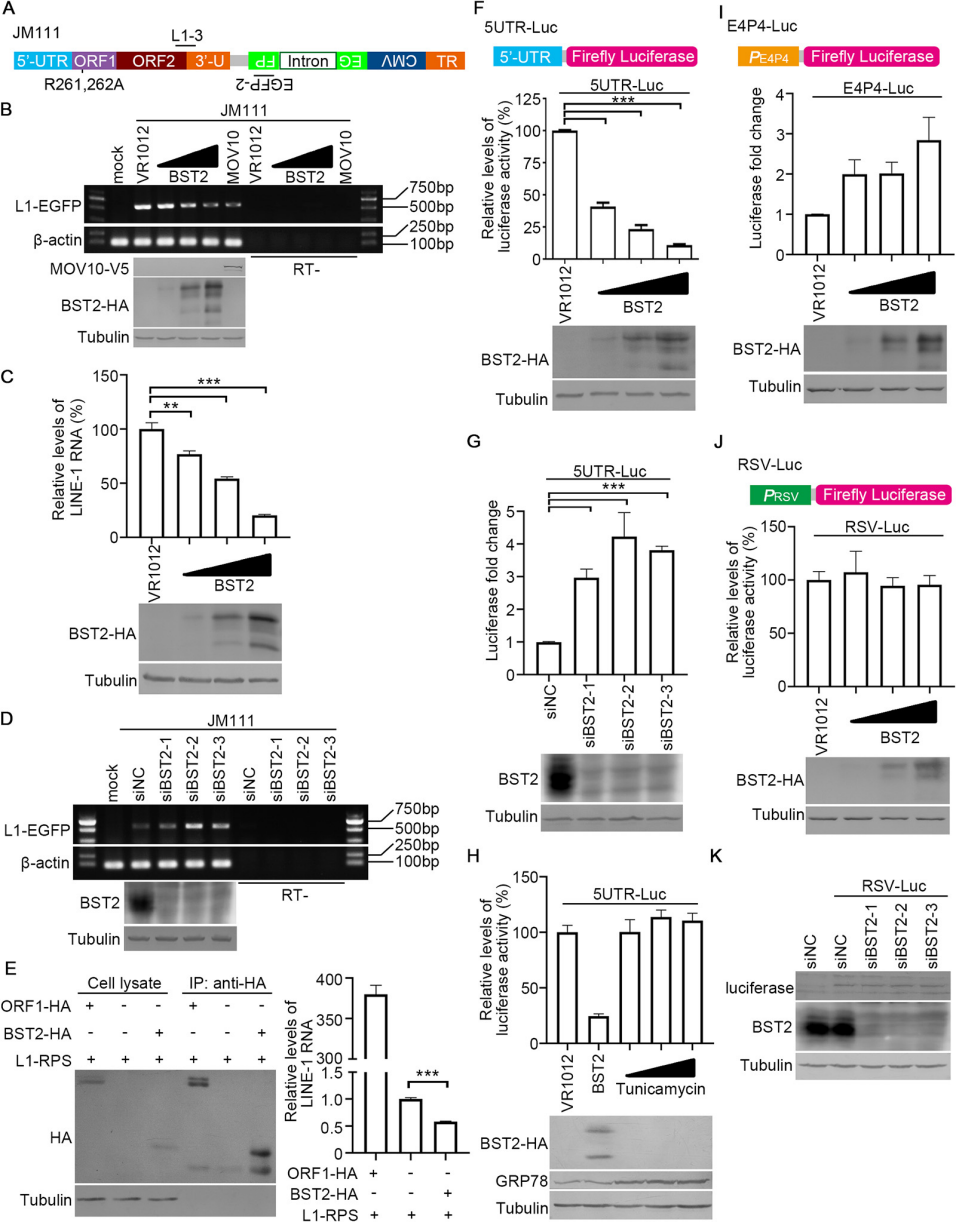
BST2 reduces LINE-1 RNA levels by compromising the promoter activity of LINE-1 5′ UTR. (A) Diagram showing the target sites for primers L1-3 forward (L1-3F) and EGFP-2 forward (EGFP-2F) on the JM111 sequence. (B) Exogenous BST2 reduces the levels of LINE-1 RNAs transcribed from JM111. MOV10 was introduced as a positive control for the reduction of LINE-1 RNA levels (53, 59). (C) Exogenous BST2 reduces the level of endogenous LINE-1 RNA in HEK293T cells. (D) Reducing endogenous BST2 expression increases LINE-1 RNA in HeLa cells. (E) BST2 does not interact with LINE-1 RNA. (F) Schematic of pGL3-5′-UTR-Luciferase (5UTR-Luc) vector. Exogenous BST2 inhibits the promoter activity of LINE-1 5′ UTR. (G) Reducing endogenous BST2 expression increases the promoter activity of LINE-1 5′ UTR in HeLa cells. (H) BST2 does not suppress LINE-1 5′ UTR through ER stress. Tunicamycin (0.5 μg/mL, 2 μg/mL, and 10 μg/mL) was used to induce ER stress in HEK293T cells, which was monitored by examining the endogenous levels of GRP78. (I) Schematic of pGL3-E4P4-Luciferase (E4P4-Luc) vector. BST2 elevates the activity of the E4P4 promoter. (J) Schematic of pGL3-RSV-Luciferase (RSV-Luc) vector. BST2 does not affect the activity of the RSV promoter. (K) Reducing endogenous BST2 expression does not affect the protein levels of luciferase expressed from RSV-Luc vector in HeLa cells. **, *p* < 0.01; ***, *p* < 0.001.

The LINE-1 locus produces a bicistronic RNA, with both ORF1p and ORF2p expressed from a single LINE-1 RNA (39). The simultaneous reductions in the levels of the LINE-1 proteins suggested that BST2 may compromise the stability of LINE-1 RNA. To confirm this hypothesis, we used a PCR-based assay to evaluate the levels of full-length LINE-1 RNA using the exogenous LINE-1 construct JM111, as previously reported (5) (Fig. 4A). Using a slightly modified assay (see Materials and Methods for details), we found that exogenous BST2 effectively reduces LINE-1 transcript levels in HEK293T cells (Fig. 4B). Consistently, quantitative real-time PCR (qRT-PCR) experiments showed that BST2 expression in HEK293T cells potently downregulates the endogenous levels of LINE-1 RNA (Fig. 4C). In contrast, a reduction in endogenous BST2 level in HeLa cells resulted in the elevation of LINE-1 RNA levels (Fig. 4D). These data support the previous observation of the effect of BST2 on ORF1p and ORF2p (Fig. 3), confirming that BST2 lowers the levels of LINE-1 proteins by reducing the levels of LINE-1 RNA.

However, further investigations revealed that BST2 did not bind to LINE-1 RNA (Fig. 4E), suggesting that BST2 may have no effect on the stability of LINE-1 RNA. Thus, we sought to determine whether BST2 compromises the synthesis of LINE-1 RNA. The 5′ UTR of LINE-1 functions as a promoter for LINE-1 RNA transcription (2). Therefore, we hypothesized that BST2 mediated LINE-1 RNA reduction by suppressing the promoter activity of LINE-1 5′ UTR. To test this hypothesis, we used a promoter assay based on firefly luciferase activity (Fig. 4F). Consistent with our hypothesis, exogenous BST2 suppressed luciferase expression driven by LINE-1 5′ UTR in HEK293T cells (Fig. 4F), whereas the inhibition of endogenous BST2 in HeLa cells increased the promoter activity of LINE-1 5′ UTR (Fig. 4G).

The expression of BST2 occurs in the endoplasmic reticulum (ER), and overexpression of BST2 has been reported to trigger ER stress (34), which in turn might influence other activities (e.g., promoter regulation) inside the cell (40, 41). To rule out this possibility, HEK293T cells transfected with 5UTR-Luc were transfected with BST2-expressing plasmid or treated with tunicamycin, which is widely used to artificially trigger ER stress (42). Endogenous levels of GRP78 were examined to confirm the presence of ER stress (43). Interestingly, treatment with tunicamycin significantly increased the endogenous levels of GRP78 but failed to regulate the promoter activity of LINE-1 5′ UTR, while BST2 potently suppressed LINE-1 5′ UTR without triggering GRP78 elevation (Fig. 4H). Taken together, these data indicate that BST2 reduces LINE-1 RNA levels by suppressing the LINE-1 5′ UTR through an ER stress-independent mechanism.

Our results showing that BST2 does not significantly influence GRP78 expression also indicated that BST2 may not have a wide effect against many promoters. To confirm this hypothesis, excluding the idea that BST2 directly affects luciferase or the possibility of BST2-specific siRNA possessing off-target effects, additional promoters such as the one from exogenous RSV or the E4P4 fragment containing endogenous CD4 enhancer and promoter were included (Fig. 4I and J). Multiple tests with BST2-expressing vector and BST2-specific siRNA indicated that neither exogenous nor endogenous BST2 suppresses the activity of the promoters (Fig. 4I to K), confirming that the BST2-mediated inhibition of 5UTR-Luc was due to BST2-mediated suppression of LINE-1 5′ UTR and not that of the luciferase gene or protein.

### BST2-induced 5′-UTR suppression is associated with BST2-mediated LINE-1 inhibition.

**FIG 5:**
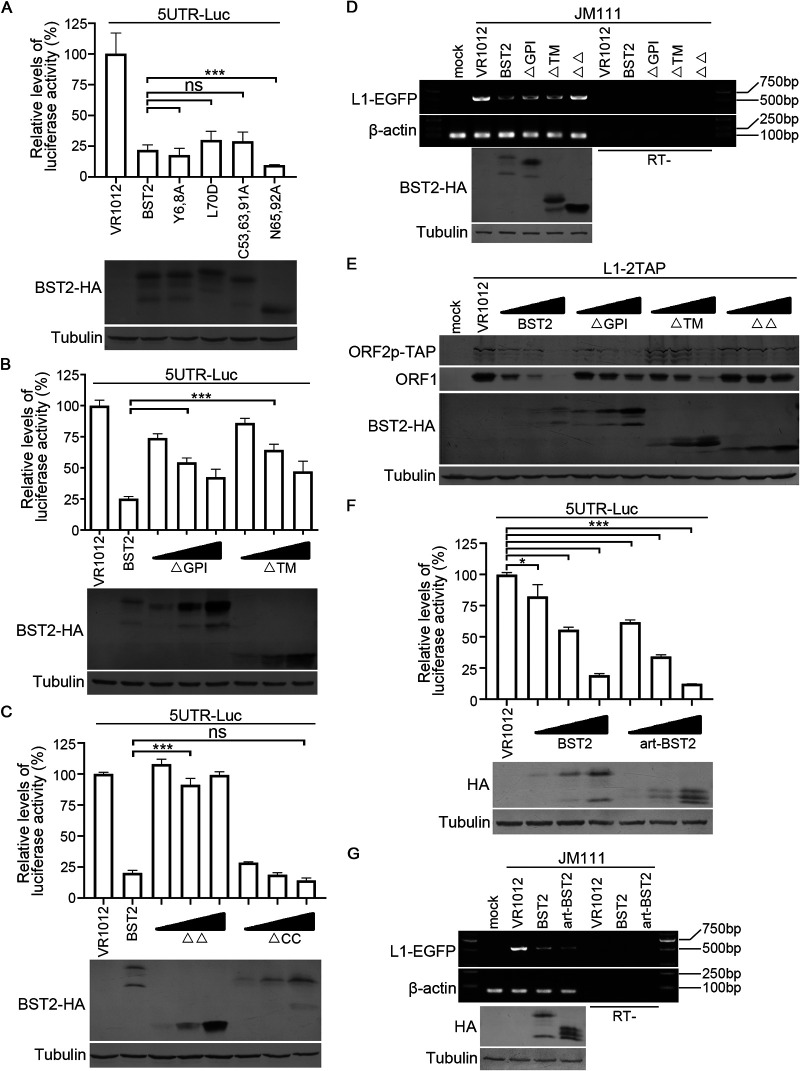
BST2-induced inhibition of 5′-UTR promoter activity contributes to BST2-mediated reduction of the levels of LINE-1 RNA and proteins as well as the activity of LINE-1 retrotransposition. (A) Tested point mutations do not compromise BST2 ability against the promoter activity of LINE-1 5′ UTR. (B) BST2 ΔGPI and ΔTM are less effective against the promoter activity of LINE-1 5′ UTR. (C) Removing both GPI and TM domains almost completely abolished BST2 potency against 5′-UTR promoter activity. (D) BST2 truncants reduce LINE-1 RNA levels to various degrees. (E) BST2 truncants suppress LINE-1 proteins levels to various degrees. (F) Art-BST2 suppresses the promoter activity of LINE-1 5′ UTR. (G) Art-BST2 reduces LINE-1 RNA levels. ns, *p* > 0.05; *, *p* < 0.05; ***, *p* < 0.001.

To validate this hypothesis and examine the association between BST2-mediated 5′-UTR regulation and LINE-1 suppression, additional tests were conducted using BST2 mutants. First, we used BST2 point mutants and found that point mutants that did not affect BST2 potency against LINE-1 replication did not affect BST2-mediated 5′-UTR regulation (Fig. 5A). Next, we used BST2 truncated mutants and found that BST2 ΔTM and ΔGPI were both weakly effective in suppressing LINE-1 5′ UTR (Fig. 5B), whereas removing both regions almost completely abrogated BST2-mediated LINE-1 5′-UTR inhibition (Fig. 5C). Moreover, truncation between the TM and GPI (i.e., ΔCC) did not affect BST2-mediated 5′-UTR regulation (Fig. 5C). Subsequently, BST2 ΔTM, ΔGPI, and ΔΔ, although to varying degrees, were found to be weakly efficient in reducing the levels of LINE-1 RNA and proteins (Fig. 5D and E). Notably, these data demonstrate the ability of these mutants to suppress LINE-1 expression (Fig. 2), confirming that BST2 suppresses the promoter activity of LINE-1 5′ UTR, which subsequently reduces LINE-1 RNA and proteins and eventually inhibits LINE-1 retrotransposition. In addition, art-BST2 was used in these tests. Consistent with its efficiency in suppressing LINE-1 (Fig. 2E), art-BST2 also inhibited the promoter activity of LINE-1 5′ UTR and decreased the levels of LINE-1 RNAs (Fig. 5F and G). This indicated that both BST2 and art-BST2 share a similar mechanism in LINE-1 suppression, revealing the possibility that proteins with similar (i.e., TM-like and GPI-like) regions may possess similar functions in 5′-UTR regulation.

### BST2-mediated LINE-1 suppression contributes to genome stabilization and innate immune regulation.

**FIG 6:**
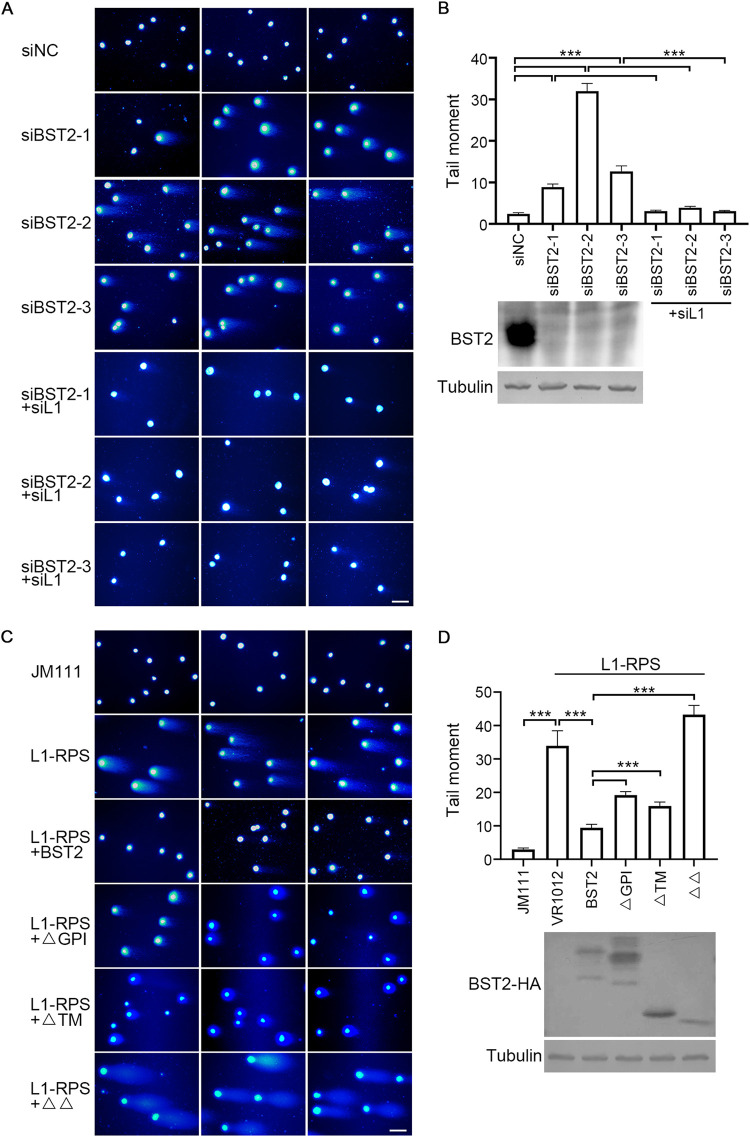
BST2 protects the genome from LINE-1-induced nicking. (A and B) Endogenous BST2 protects genome integrity in HeLa cells. Representative images are shown on the left, while the tail moment of the comets was analyzed using 100 cells for each sample with CASP software, and the Western blotting images are shown on the right. (C and D) Exogenous BST2 prevents LINE-1-induced genome damaging. Representative images are shown on the left, while the tail moment of the comets was analyzed using 100 cells for each sample with CASP software, and the Western blotting images are shown on the right. ***, *p* < 0.001. Scale bar = 20 μm.

LINE-1 retrotransposition involves a special process called target-site-primed reverse transcription (TPRT). In brief, LINE-1 RNP recognizes “AATTTT” motifs in the genomic DNA, induces a nicking between “A” and “T,” and initiates reverse transcription with the loosened DNA strand as the primer (see 44 for details). Thus, the process of TPRT offers LINE-1 the ability to nick the host genome, which, if unregulated, can result in breaks in the genomic DNA and disruption of cellular activity (13, 14). To determine whether BST2 is a potent LINE-1 suppressor that functions as a genome stabilizer, we first knocked down endogenous BST2 expression in HeLa cells and checked for DNA damage using a single cell gel electrophoresis assay (also known as the COMET assay). As shown in Fig. 6A and B, DNA damage was readily detected in HeLa cells treated with siBST2 for 48 h, which was abolished by co-transfection with LINE-1-specific siRNA, suggesting that the observed instability of genomic DNA was induced by the elevated activity of endogenous LINE-1 due to compromised BST2 expression. To further explore the role of LINE-1 in the above phenomenon, HEK293T cells were transfected with exogenous L1-RPS, along with a control or BST2-expressing vector. At 96-h posttransfection, DNA damage was observed in cells transfected with L1-RPS but not in cells co-transfected with L1-RPS and BST2-expressing vectors (Fig. 6C and D). In addition, BST2 mutants, including ΔTM, ΔGPI, and ΔΔ, which were weakly effective in regulating LINE-1 retrotransposition, were also weakly potent in preventing LINE-1-induced DNA damage (Fig. 6C and D). These data suggest that, by suppressing LINE-1 activity, BST2 maintains genomic integrity.

**FIG 7:**
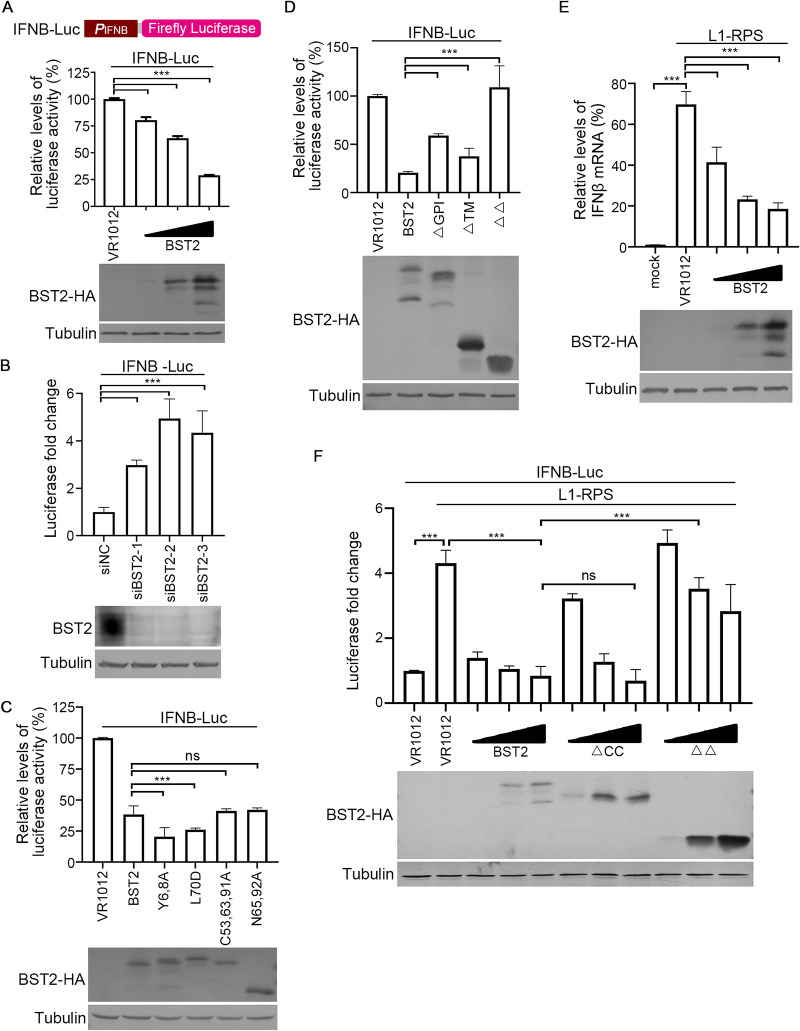
BST2 suppresses LINE-1-triggered innate immune activation. (A) Schematic of pGL3-IFNB-Luciferase (IFNB-Luc) vector. Exogenous BST2 suppresses the activation level of the innate immune system. (B) Endogenous BST2 inhibits innate immune activation in HeLa cells. (C) Tested point mutations do not compromise BST2 activity in suppressing the innate immune system. (D) BST2 truncants suppress innate immune activation to various degrees. (E) BST2 suppresses LINE-1-triggered endogenous innate immune activation in HEK293T cells. (F) BST2 suppresses LINE-1-triggered exogenous innate immune activation. ns, *p* > 0.5; ***, *p* < 0.001.

In our previous study, LINE-1 RNP has been identified as an endogenous trigger of the RNA-sensing pathway, which results in the activation of the innate immune system and production of IFN (16). Therefore, we hypothesized that BST2-mediated reduction of LINE-1 RNA and proteins (through 5′-UTR suppression) would be accompanied by decreased levels of LINE-1 RNP, which would then lead to the regulation of the innate immune system. Consistently, a luciferase-based promoter activity assay indicated that BST2 expression lowered the activity of the *IFNB* promoter in HEK293T cells (Fig. 7A), whereas a reduction in the endogenous BST2 levels increased luciferase expression driven by the *IFNB* promoter in HeLa cells (Fig. 7B). Additional tests confirmed that BST2 mutants that were capable of suppressing LINE-1 were also potent in regulating the activation of the innate immune system in HEK293T cells (Fig. 7C). In contrast, mutants including ΔTM, ΔGPI, and ΔΔ were less efficient in suppressing the *IFNB* promoter (Fig. 7D), correlating with their effects on LINE-1 inhibition. To further explore the involvement of LINE-1 in BST2-mediated *IFN* regulation, the exogenous LINE-1 vector, L1-RPS, was used to activate the innate immune system in HEK293T cells (Fig. 7E and F). Again, BST2 potently reduced the activity of the endogenous and exogenous *IFNB* promoter, which was elevated by exogenous LINE-1 (Fig. 7E and F). Notably, BST2 ΔΔ was almost completely incapable of suppressing LINE-1-induced innate immune activation, whereas ΔCC retained full efficacy (Fig. 7F). Thus, BST2 functions as an innate immune regulator via LINE-1 regulation.

## DISCUSSION

Although retroelements are considered fossils of ancient retroviral insertions, the replication of LINE-1, which is the only type of autonomous retroelement in human cells, does not involve virion budding and release. Therefore, it was surprising to find that BST2, which suppresses the release of HIV virions by tethering them physically on the surface of producer cells, functions as a potent LINE-1 suppressor. Different mechanisms are used in BST2-mediated suppression of HIV and LINE-1. In this study, first, we confirmed that BST2 is an effective LINE-1 suppressor. Next, we demonstrated that BST2 indeed utilizes different mechanisms to target HIV and LINE-1, by showing that some BST2 mutants that have been reported incompetent in HIV suppression maintained their efficiency in LINE-1 inhibition. Further investigations indicated that BST2 inhibits the promoter activity of LINE-1 5′ UTR, which leads to the reduction of LINE-1 RNA and proteins and ultimately to the inhibition of LINE-1 replication. Interestingly, studies with BST2 truncation mutants have suggested that the CT domain, which is the only part of BST2 facing the cytoplasm, is dispensable in BST2-mediated suppression of LINE-1 5′ UTR. Moreover, the two membrane-associated domains of BST2, TM and GPI, are both important for BST2-mediated repression of the promoter activity of LINE-1 5′ UTR. Removing both regions almost completely abolished BST2-mediated LINE-1 retrotransposition. Results of experiments using art-BST2 suggested that the structures, rather than the sequences, of TM and GPI are important for BST2 to inhibit LINE-1 5′ UTR. Therefore, our data suggest that BST2 regulates LINE-1 promoter activity through a structure-dependent mechanism, which is critical for BST2-mediated LINE-1 suppression.

BST2 alters gene expression through its CT domain to activate the NF-κB signaling pathway (33, 34). However, point mutations and deletions indicated that NF-κB pathway activation and the CT domain are not involved in the BST2-mediated suppression of LINE-1. Intriguingly, the two membrane-associated domains of BST2, TM and GPI, are both important for BST2-mediated 5′-UTR regulation, and removing these regions almost completely abrogated BST2-mediated suppression of LINE-1 retrotransposition. BST2 is expressed in the rough endoplasmic reticulum (RER) and modified in the RER and Golgi apparatus before being transported to the cell surface. Notably, ER and Golgi have been reported to interfere with host gene expression through secretory stress signaling (45, 46). The presence of BST2 may trigger this signaling pathway and suppress the transcription of LINE-1. Alternatively, BST2 may act on the cell surface where different receptors are located. These receptors are normally activated by extracellular stimuli and initiate downstream pathways that lead to changes in host gene expression via promoter regulation. It is therefore possible that BST2 interacts with and activates such receptors, resulting in the regulation of gene promoters including LINE-1 5′ UTR. Although less likely, BST2 may bind to a transcription factor with its TM and GPI regions and restrict the factor from being processed in or released from the ER and/or Golgi, sabotaging the assembly of the transcriptional machinery that would have formed at LINE-1 5′ UTR. Both mechanisms are structure-dependent, as they could be performed by the art-BST2 protein that shares high topology similarity but lacks sequence homology with wild type BST2. However, the effect appears to differ from a promoter to another, as BST2 does not suppress RSV and E4P4 promoters. Thus, the result of our study on the mechanism of BST2-mediated LINE-1 suppression suggests that BST2 alters the activity of certain promoters via its membrane-associated TM and GPI domains, which may influence the expression of host genes to alter the activity of the cell or viral genes to compromise viral replication.

Activation of the innate immune system has recently been associated with LINE-1 retrotransposition. Intriguingly, LINE-1 triggers innate immune activation through multiple pathways. Stetson et al. (47) suggested for the first time that changes in cytosolic levels of LINE-1 DNA fragments contribute to the activation of the innate immune system (47). This indicates that, although LINE-1 cDNA is part of the genomic DNA, LINE-1 TPRT may cause its removal from the genome, possibly because of host repair mechanism(s) acting at the nicking point, and release it into the cytoplasm where the LINE-1 DNA may trigger the activation of DNA sensors. We recently demonstrated that the formation of LINE-1 RNP allows LINE-1 RNA to trigger IFN production through MDA5- and RIG-I-mediated RNA-sensing pathways (16). Notably, the innate immune system responds to changes in LINE-1 RNA levels rapidly, because altering cytoplasmic LINE-1 DNA levels requires a long period to allow additional steps, such as TPRT. Based on the above observations, we hypothesized that many LINE-1 inhibitors may also function as innate immune suppressors through LINE-1 regulation. By suppressing the promoter activity of LINE-1 5′ UTR, BST2 inhibits the formation of LINE-1 RNP by reducing the levels of LINE-1 RNA and proteins, which further decreases the generation of LINE-1 DNA due to few TPRT events. Consistently, BST2 lowered the activation of the *IFNB* promoter in the presence of exogenous LINE-1. Notably, BST2 mutants that fail to inhibit LINE-1 activity are also incompetent in regulating the innate immune system. Therefore, BST2 suppresses innate immune activation by compromising LINE-1 retrotransposition, which supports our hypothesis that LINE-1 suppressors function as innate immune regulators (16).

Additionally, innate immune regulation is not the only outcome of BST2-mediated LINE-1 suppression. LINE-1 replication is known to cause genomic DNA damage, most likely via LINE-1 TPRT (13, 14). Nicks caused by LINE-1 influence gene expression and genomic stability, as the introduction of exogenous LINE-1 reportedly results in cell cycle arrest and genomic DNA breaks. Similar occurrences have been detected in cells where the levels of LINE-1 suppressors were downregulated, as previously observed with TREX1 (25) or BST2 in this study. In other words, by suppressing LINE-1 retrotransposition, BST2 protects the integrity of genomic DNA. Further, genomic damage and breaks have been recently linked to innate immune activation (48). Thus, preventing LINE-1-induced DNA damage not only endows BST2 with a new function as a genome stabilizer but also strengthens its role as a regulator of innate immunity.

BST2 is a restrictive factor that suppresses HIV and LINE-1 through distinct mechanisms. Other examples of such factor include APOBEC3, ADAR1, SAMHD1, and TREX1. For instance, the catalytic activities of SAMHD1 and TREX1, as dNTPase and DNA exonuclease, respectively, target the process or product of reverse transcription, which is the mechanism by which both proteins act on HIV. However, the catalytic activities of SAMHD1 and TREX1 do not affect LINE-1 replication. Instead, SAMHD1 and TREX1 potently reduce the protein levels of ORF2p and ORF1p, respectively (22, 25). In addition, SAMHD1 restricts ORF1p to stress granules (23). By acting on LINE-1 proteins, SAMHD1 and TREX1 compromise the formation and localization of LINE-1 RNP. Similar scenarios have also been observed with APOBEC3 proteins and ADAR1, which inhibit LINE-1 retrotransposition through interaction with LINE-1 proteins and RNAs, respectively (49, 50), instead of using their catalytic activities while acting on HIV (18, 51). As mentioned above, LINE-1 triggers innate immune activation in several steps, whereas the formation of LINE-1 RNP not only activates RNA sensors such as MDA5 and RIG-I but also initiates TPRT, which results in the activation of DNA sensing pathways. Accordingly, compromising the formation, function, and/or localization of LINE-1 RNP is the best approach to fully suppress LINE-1-induced innate immune activation, which is the mechanism used by other restrictive factors as recently summarized (52) and by BST2 in this study. No direct involvement of the plasma membrane or membrane-associated structure was observed during LINE-1 replication, excluding the possibility that BST2 uses the same tethering mechanism while acting on HIV and LINE-1. Instead, BST2 targets the first step of LINE-1 retrotransposition: transcription. By suppressing the LINE-1 5′ UTR, BST2 potently decreases the generation of LINE-1 RNAs and proteins that are essential for the formation of LINE-1 RNP. Additionally, the mechanism of BST2-mediated LINE-1 suppression further confirms our notion that reducing the formation and/or integrity of LINE-1 RNP is a common feature, although this sometimes requires an alternative mechanism involving host restriction factors that also function as LINE-1 suppressors.

## MATERIALS AND METHODS

### Cell culture and transfection.

HEK293T and HeLa cells were cultured in Dulbecco’s modified Eagle’s medium (Gibco, C11995500BT) with 10% fetal bovine serum (Biological Industries, 04-001-1ACS) and Pen-Strep (Biological Industries, 03-031-1B). BST2 and LINE-1 specific siRNA were transfected into HeLa cells using Lipofectamine RNAiMAX (Invitrogen, P/N56532) reagent. Plasmids were transfected with Lipofectamine 3000 (Invitrogen, L3000015) reagent in HeLa cells or polyethylenimine (Polysciences, 23966-2) in HEK293T cells. BST2-expressing vectors (25 ng, 75 ng, or 225 ng, including both wild type and mutants) were used per well when transfected into HEK293T and HeLa cells in a dose-dependent manner, otherwise 250 ng was used per well. All transfections were performed with Opti-MEM (Gibco, 802679) according to the manufacturer’s protocol.

### Plasmids and synthetic oligonucleotides.

The retrotransposition-competent vector 99 PUR RPS EGFP (L1- RPS) (36), the retrotransposition-incompetent 99 PUR JM111 EGFP (JM111) (36), pc-L1-1FH (L1-1FH) (53), VR1012 (54), VR1012-ORF1-Myc (ORF1-Myc) (25), VR1012-MOV10-V5 (53), pGL3-IFNB-Luciferase (IFNB-Luc) (55), pGL3-5′-UTR-Luciferase (5UTR-Luc) (25), VR1012-BST2-HA, BST2 mutations (Y6,8A; L70D; C53,63,91A; and N65,92A), and truncants (ΔGPI, ΔTM, and ΔCT) (29) have been described previously. The pGL3-E4P4-Luciferase (E4P4-Luc) and pGL3-RSV-Luciferase (RSV-Luc) were kindly provided by Dr. Xiao-Fang Yu. VR1012-EGFP (VR-EGFP), VR1012- ORF1-HA (ORF1-HA), VR1012- TREX1-HA (TREX1-HA), and BST2 ΔGP1 ΔTM-HA (ΔΔ) were constructed into VR1012. The VR1012-BST2 ΔCC-HA (ΔCC) was constructed using primers combined with the sequence before and after coiled-coil coding region, thus forming a loop on the template to truncate the coiled-coil domain of BST2. VR1012-artificial-BST2-HA (art-BST2) was constructed as previously described (31). Synthetic BST2-specific siRNA (siBST2) and LINE-1-specific siRNA (siL1) were purchased from RiboBio, with the following sequences: siBST2-1: (5′-GAATCGCGGACAAGAAGTA-3′); siBST2-2: (5′-CCTTGATTATCTTCACCAT-3′); siBST2-3: (5′-GAGAGATCACTACATTAAA-3′); siL1: (5′-TGAGCAAAGCCTCCAAGAA-3′). The pc-L1-2TAP (L1-2TAP) was constructed through DNA recombination using the pEASY-Basic Seamless Cloning and Assembly Kit (Transgen, China), according to the manufacturer’s instructions. Three fragments were amplified from the L1-RPS. Fragment 1 contained the sequence from the beginning of the 5′ UTR to the end of ORF2, while fragments 2 and 3 were the 3′-UTR fragments flanking the antisense EGFP expression cassette in the L1-RPS fragment. Another DNA fragment (fragment 4, which encodes the TAP tag) was synthesized by Generay Biotech Co., Ltd (Shanghai, China), according to the sequence from the pMSCV-TAP plasmid (56). All fragments were then inserted into the pcDNA6/myc-His B vector (Invitrogen, CA) through DNA recombination in the order 1-4-2-3 to generate L1-2TAP, containing a complete L1_RP_-based LINE-1 sequence that expresses a TAP-tagged ORF2p.

### Antibodies and reagents.

The following antibodies were used in this study: anti-tubulin (TransGen, HC101-02), anti-HA (Biolegend, 901513), anti-Myc Tag, clone 4A6 (Millipore, 05-724), anti-LINE-1 ORF1p, clone 4H1 (Millipore, MABC1152), anti-BST2 (Proteintech, 13560-1-AP), anti-TAP tag (Thermo Scientific, CAB1001), anti-luciferase (Proteintech, 67293-1-lg), anti-GRP78 (Wanlei, WL03157), and anti-V5 (Invitrogen, 46-0705). All antibodies were used according to the manufacturers’ protocols. Tunicamycin (Abcam, ab120296) was used to induce ER stress.

### LINE-1 retrotransposition assays.

The LINE-1 retrotransposition assay was performed as previously described (22, 36). L1-RPS is based on natural LINE-1, with an antisense EGFP reporter cassette in the 3′ UTR, while the *EGFP* gene is interrupted by a sense Group I intron. EGFP can only be detected when the LINE-1 transcript is spliced and reverse transcribed, its cDNA is inserted into the host genome, and the *EGFP* reporter gene is expressed from its own CMV promoter. JM111 is similar to L1-RPS, which contains 2-point mutations in ORF1p that completely abolish retrotransposition. Briefly, L1-RPS or JM111 was transfected into HEK293T cells or HeLa cells at 1 μg in 24 well plates, together with VR1012 or one of the test plasmids. The cells were tested via flow cytometry using FACSCalibur 96 h after transfection. Gating exclusions were based on the background fluorescence of the plasmid JM111. A total of 20,000 single-cell events per sample were collected and analyzed using FlowJo (version 7.6.1).

### Quantitative real-time reverse transcription-PCR (qRT-PCR).

Total RNA from samples of interest was extracted using the FastPure Cell/Tissue Total RNA isolation kit (Vazyme, RC101, containing DNase treatment) and then subjected to reverse transcription with MonScipt^TM^ RTIII All-in-One Mix (Monad, RN05004M, including DNase treatment before reverse transcription). An RT control (without reverse transcriptase) was prepared for each test and used as a parallel sample to detect any possible contamination of genomic DNA (data not shown). qRT-PCR experiments were performed using MonAmp ChemoHS qPCR Mix (Monad, RN04001N) and specific primers. The reactions were performed under the following conditions as suggested by the manufacturer: 94°C for 30 s, then 40 cycles at 94°C for 10 s, and 60°C for 30 s, followed by a dissociation protocol. Single peaks in the melting curve analysis indicated specific amplicons. *ACTB* mRNA was monitored as a cellular mRNA control (data not shown). The primers used were: L1-3, forward (5′-CAAACACCGCATATTCTCACTCA-3′) and reverse (5′-GCTGATATGAAATTCTGGGTTGA-3′); *ACTB*, forward (5′-ACCGAGCGCGGCTACAG-3′) and reverse (5′-CTTAATGTCACGCACGATTTCC-3′).

### PCR assay.

The above-stated primers were also used in the PCR assay, which was used to confirm the effect of BST2 on full-length LINE-1 RNA, with the help of the JM111 plasmid and minor modification of a previously reported protocol (25). Briefly, 1 μg JM111 and BST2-expressing vectors (25 ng, 75 ng, and 225 ng)/MOV10 expressing vectors (250 ng) were co-transfected into HEK293T cells, or HeLa cells were transfected with BST2-specific siRNA, followed by 1 μg JM111 transfection at 24-h posttransfection. Cells were then subjected to RNA extraction and reverse transcription 48-h posttransfection. PCR based on synthesized cDNA was performed using a 2× Phanta Max Master Mix (Vazyme, P515). The reactions were performed under the following conditions as suggested by the manufacturer: 95°C for 3 min, then 30 cycles at 95°C for 15 s, 56°C for 15 s, and 72°C for 90 s, followed by 72°C for 5 min.

Levels of *ACTB* mRNA (as a cellular mRNA control) were monitored using the primer pairs. Levels of JM111 RNA were detected using L1-3 forward and *EGFP*-2 forward (5′-ACTACCTGAGCACCCAGTCC-3′). Thus, the amplicon covered the regions of both LINE-1 and EGFP cassette. The antisense EGFP cassette in JM111 has its own poly A signal; thus, the transcription of *EGFP* mRNA does not contain a LINE-1 fragment. However, the *EGFP* gene does not exist in the human genome. Therefore, the LINE-1 part ensures that the amplicon represents LINE-1 RNA transcribed from the 5′ UTR of JM111 instead of the antisense CMV promoter, while the EGFP part prevents possible contamination from endogenous LINE-1 DNA/RNA. RT- control for each sample was also included to exclude possible contamination of JM111 DNA. Thus, the amplicon based on L1-3 forward and *EGFP*-2 forward could represent full-length LINE-1 RNA transcribed from JM111.

### Co-immunoprecipitation.

Co-immunoprecipitation(co-IP) experiments were performed as previously reported (57). HEK293T cells were transfected with TREX1 (400 ng), BST2 (1 μg), and/or ORF1p expressing vectors (1,200 ng for TREX1-transfected cells and 400 ng for others), and then harvested at 48-h posttransfection. Samples were washed with 1× PBS, suspended in lysis buffer (50 mM Tris-HCl [pH 7.5], 150 mM NaCl, and 0.5% NP-40, supplemented with cOmplete™ Tablets EDTA-free EASYpack [Roche]), sonicated at 15% power for 15 × 3 s breaks separated by 3-s intervals, and then centrifuged at 12,000 rpm for 10 min to harvest the supernatant. Input samples were incubated with anti-HA Magnetic Beads (Thermo Pierce, 88837) overnight and then washed six times with wash buffer (20 mM Tris-HCl [pH 7.5], 100 mM NaCl, 0.1 mM EDTA, and 0.05% Tween 20). The samples were then eluted with 50 mM glycine-HCl (pH 2.5) and subjected to the following tests.

### Luciferase reporter assay.

The TransDetect Single-Luciferase Reporter assay kit (TransGen, FR101-01) was used to check whether BST2 affected the promoter activity of the LINE-1 5′ UTR and the *IFNB* promoter. Briefly, 200 ng 5UTR-Luc/IFNB-Luc/E4P4-Luc/RSV-Luc- and BST2-expressing vectors were transfected into HEK293T cells. At 48-h posttransfection, luciferase activity was detected according to the manufacturer’s protocol. Readings of pGL3-transfected sample were used to remove background noise and are not shown.

### Comet assays.

Comet assays were conducted according to previously published procedures (14, 25, 58). Briefly, HEK293T cells were transfected with L1-RPS along with the vectors expressing wild type BST2, mutants, and truncants, or the control vector. Alternatively, HeLa cells were treated with a BST2-specific siRNA. At 96-h posttransfection for HEK293T cells (or 48 h for HeLa cells), the cells were harvested and washed with 1×PBS, mixed with 0.5% low-melting agarose (Agarose LMP, Sangon Biotech, B0015) at 37°C, placed on a precleaned microscope slide already covered and preoverdried with 0.5% normal melting agarose (Regular Agarose G-10, Biowest, CB005) and immediately covered with a cover glass, and kept at 4°C for 5 min. After removing the cover glass gently, the slide was covered with a third layer of low-melting agarose using another cover glass, which was removed after being horizontally placed at 4°C for another 5 min. The solidified agarose was then immersed in a lysing solution (1% sodium sarcosinate, 2.5 M NaCl, 100 mM Na_2_-EDTA, 10 mM Tris [pH 10.0], 1% freshly added Triton X-100, and 10% DMSO) for 1 h to release and unfold the DNA, followed by immersion in an electrophoretic buffer (1 mM Na_2_-EDTA and 300 mM NaOH [pH 10.0]) for 20 min. Electrophoresis was conducted at 25 V for another 20 min. The slide was subsequently washed with 0.4 M Tris-HCl (pH 7.5) twice and then with deionized water twice. Finally, agarose on the slides was stained with Fluoroshield^TM^ with DAPI (Sigma, F6057), covered with a glass cover, and observed under an Olympus IX51 inverted microscope. The tail moment of the comets was measured for at least 100 cells for each sample using CASP1.2.3 beta1.

### Quantification and statistical analysis.

Flow cytometry data are presented as the mean ± standard deviation of three technical replicates within one experiment, and other data are presented as means ± standard error of the mean of at least three independent biological replicates. Data were analyzed using unpaired two-tailed Student's *t*-tests. Analyses were performed using SPSS 17.0 and GraphPad Prism 8.0.2 (263). Results are presented as not significant (ns) at *p* > 0.05 or significant at levels *, *p* < 0.05; **, *p* < 0.01; and ***, *p* < 0.001.
